# SDF-1*α* Facilitates Mesenchymal Stem Cells to Induce Regulatory B Cell Differentiation from Patients with Immune Thrombocytopenia

**DOI:** 10.1155/2021/3254488

**Published:** 2021-11-08

**Authors:** Zhe Chen, Shufen Zhou, Jianyun Li, Hui Li, Can Huang, Qin Guo, Tiantian Zhang, Bingya Yang, Chuanqing Tu, Chengshan Guo

**Affiliations:** ^1^Department of Rheumatology and Immunology, Southern Medical University Affiliated Shenzhen Baoan Hospital, Shenzhen, China; ^2^Department of Rheumatology and Immunology, Guangdong Medical University Shenzhen Baoan Clinical College, Shenzhen, China; ^3^Department of Rheumatology and Immunology, Shenzhen University Second Affiliated Hospital, Shenzhen, China; ^4^Department of Hematology, Guangdong Medical University Shenzhen Baoan Clinical College, Shenzhen, China; ^5^Spunolin Biotechnology Co., Ltd., Shenzhen, China

## Abstract

B cells play a central role in the pathogenesis of immune thrombocytopenia (ITP) by participating in humoral immunity. Meanwhile, regulatory B cells (Bregs), one subset of B cells, express negative regulatory effect on ITP. Mesenchymal stem cells (MSCs) have been demonstrated in the ability to induce immunosuppression, and stromal cell-derived factor-1*α* (SDF-1*α*) plays an important role in the migration and survival of MSCs. To investigate the mechanism of SDF-1*α* in controlling umbilical cord-derived MSCs (UC-MSCs) in inducing regulatory B cell differentiation of patients with ITP, we reconfirmed that SDF-1*α* promotes the proliferation of MSCs at the low doses of 0.05 *μ*g/mL and 0.1 *μ*g/mL but inhibits the proliferation and promotes the apoptosis of UC-MSCs at the high doses 0.5 *μ*g/mL and 1 *μ*g/mL; when UC-MSCs are cocultured with SDF-1*α* at 0.1 *μ*g/mL, the decreased proportion of CD19^+^/CD24^hi^/CD38^hi^ cells and IL-10-producing B cells (B 10 cell), considered as the Breg subset from ITP significantly enhanced, and the content of IL-10 in the supernatant is also obviously increased. The proportion of Bregs and the IL-10 secretion could be further promoted by the UC-MSCs treated with 0.1 *μ*g/mL SDF-1*α*, which could also promote the miRNA-133 expression of UC-MSCs in an exosome-dependent manner; moreover, while the UC-MSCs were transfected with the miR-133 inhibitor, the proportion of induced Bregs decreased obviously when cocultured with peripheral blood mononuclear cells (PBMCs) of ITP. We conclude that UC-MSCs could effectively enhance the decreased proportion of Bregs from ITP; at appropriate concentrations, SDF-1*α* may promote the proliferating and survival ability of UC-MSCs and improve the production of Bregs induced by UC-MSCs through controlling miRNA-133 expression in the exosomes.

## 1. Introduction

Immune thrombocytopenia (ITP) is an immune-mediated acquired disease characterized by a persistent or transient decrease of platelet count. Traditionally, B cells have been thought to contribute to the pathogenesis of this autoimmune disease through platelet-specific autoantibody production. In fact, B cells play a central role in the pathogenesis of ITP, because it not only generates autoantibody participating humoral immunity but also acts as antigen presenting cells (APCs) to enhance immune reaction and even directly secretes some cytokines leading to platelet destruction [1]. However, there is another subset capable of negative immune regulation named regulatory B cells (Bregs) to maintain the immune homeostasis under normal physiological conditions or in autoimmune diseases [2, 3]. Unfortunately, Bregs were found expressing dysregulation in ITP [4], so promoting the differentiation and expanding the role of Bregs in immunoregulation is very important. Mesenchymal stem cells (MSCs) have been investigated in cell-based therapies because of the remarkable regenerative properties and the ability to induce immunosuppression via interaction with cells from both the innate and adaptive immune systems [5–11]. It is very interesting that MSCs can induce Bregs and regulatory T cell (Treg) production [12–15], as well as suppress the effector B cells [16–18]. However, the mechanism of MSCs in inducing Bregs of ITP still remains unclear, and in particular, there still remain many difficulties for MSCs in clinical treatment because of the obstacles in migration and survival.

Stromal cell-derived factor-1*α* (SDF-1*α*) is a well-known major chemotactic factor induced by hypoxia and ischemic tissue and is critical for the process involving MSC organ-specific homing in injury tissue through interaction with its cognate receptor CXC chemokine receptor 4 (CXCR4) on the surface of MSCs [19–21]. Furthermore, it is valuable to explore whether SDF-1*α* is involved in the immunoregulation of MSCs, especially the mechanism in inducing Breg differentiation. The immunological properties of MSC exosomes to modulate the immune system have been provided [22]. As miR-133 has been shown to influence the MSC function [23], we thus hypothesized that SDF-1*α* may change the miR-133 expression and enhance the ability of MSCs to promote Bregs by means of exosomes. Due to noninvasive and easy access of the tissue origins from human delivery wastes when getting the umbilical cord (UC), the UC derived-MSCs (UC-MSCs) exhibit a higher degree of multipotency and a stronger ability to self-renew than bone marrow-derived MSCs (BM-MSCs) or adipose tissue-derived MSCs (AD-MSCs) [7, 8], and we used UC-MSCs to investigate their effect on inducing Breg differentiation ITP and the mechanism of SDF-1*α* in controlling proliferation, apoptosis, and regulatory function of UC-MSCs and proved that SDF-1*α* promotes the expression of miR-133 in MSCs to facilitate their ability in an exosome-dependent manner. The aim of the research is to provide a potential approach in the management of ITP.

## 2. Materials and Methods

### 2.1. Patients and Controls

Between January 2019 and June 2020, 18 patients (7 males and 11 females, age range 19–71 years, median age 29.5 years) with active ITP in the Shenzhen Baoan Hospital affiliated to the Southern Medical University and Guangdong Medical University Shenzhen Baoan Clinical College were enrolled. All of the cases fulfilled the diagnosis criteria of ITP as previously described [24, 25], and the inclusion criteria are in accordance with these diagnosis criteria. Patients' platelet counts ranged from 1 to 74 × 10^9^/L, with a median count of 13 × 10^9^/L. None of them had received any corticosteroid or immunosuppressive therapy within the 3 months prior to sampling. Patients with diabetes, hypertension, cardiovascular diseases, pregnancy, active infection, or connective tissue diseases, such as systemic lupus erythematosus, were excluded. The parallel consisted of 10 healthy adult volunteers (2 males and 8 females, age range 20–55 years, median 31.0 years), whose platelet counts ranged from 136 to 393 × 10^9^/L, with the median count of 227 × 10^9^/L.

### 2.2. UC-MSC Isolation, Culture, and Differentiation

Wharton's Jelly from the umbilical cord was cut into 1 mm^3^ pieces and digested with 1 mg/mL collagenase II (Sigma, USA) diluted in PBS at 37°C for 9 h. The acquired cell suspension was washed with PBS for three times and seeded in Dulbecco's modified Eagle's medium (DMEM) with 10% fetal calf serum (Gibco-BRL, USA), 2 mmol/L glutamine, antibiotics (100 U/mL penicillin, 100 mg/mL streptomycin), and 10 ng/mL basic fibroblast growth factors (Gibco-BRL). The cells were cultured at 37°C in an atmosphere of 5% CO_2_. The adherent cells were cultured to confluence. Culture medium was changed every week, and the cells were treated with 0.25% trypsin (Gibco-BRL) for subculture. The cells (3 × 10^5^) at passage 3 (Supplementary 1A) were seeded in 12-well plates and cultured to reach about 90% confluence for identifying the differentiation property of the cells from UC [26–28]. Briefly, for the osteogenic differentiation medium: DMEM complete medium containing 10 nM dexamethasone, 100 *μ*M l-ascorbic acids, 10 mM *β*-glycerophosphate I, and 5% FBS; for adipogenic differentiation medium: DMEM complete medium containing 0.5 mM 3-isobutyl-1-methylxanthine, 60 *μ*M indomethacin, 10 nM dexamethasone, 10 *μ*g/mL insulin, and 5% FBS. Oil Red O was used for detection of differentiation potency to adipocytes and alkaline phosphatase staining for detection of differentiation potency to osteocytes (Supplementary 1B–1C). The staining results were analyzed with a microscope.

### 2.3. FACS Analysis

The phenotypes of UC-MSCs incubated with labelled antibodies were analyzed using FACSCalibur (BD Biosciences, USA), positive cells were counted, and the signals for the corresponding immunoglobulin isotypes were for contrasts (Supplementary 1D). The antibodies included FITC-labelled anti-CD44, PE-labelled anti-CD105, PC-labelled anti-CD29, APC-labelled anti-CD34, and APC-cy7-labelled anti-CD45 (eBioscience, CA, USA). The markers of Bregs were detected by the following antibodies: CD19, CD24, CD38, and IL-10 (eBioscience, CA, USA).

### 2.4. MSC Viability and Apoptosis Assays

Referring to the method described [29], MSCs (2.0 × 10^5^) were seeded in 24-well plates with medium containing SDF-1*α* (Sigma, USA) in different concentrations (0 *μ*g/mL, 0.05 *μ*g/mL, 0.1 *μ*g/mL, 0.5 *μ*g/mL, 1.0 *μ*g/mL). Cell proliferation was measured from 0 to 4 days successively using a 3-(4,5-dimethylthiazol-2-yl)-2,5-diphenyltetrazolium bromide (MTT, Sigma, USA) assay. MSC apoptosis was assessed using Annexin V-APC-Propidium Iodide (Annexin V/PI, Sproutbios, Shenzhen, China) staining with FACS after 48 h. Importantly, we could select an appropriate concentration of SDF-1*α* to promote the viability and immunoregulation function of MSCs at this experimental procedure.

### 2.5. B Cell Isolation

Peripheral blood mononuclear cells (PBMCs) were isolated by Ficoll-Hypaque gradient centrifugation (Haoyang, China). B cells were selected by the magnetic-activated cell sorting (MACS) cell separation system (Miltenyi Biotec, Germany) using anti-CD19 microbeads. Briefly, PBMCs were incubated for 30 min at room temperature with the microbeads conjugated with anti-CD19 antibody. Then, the incubated PBMCs were transferred into the LS column placed on the magnetic separator. The unlabelled cells would be discarded after passing through the magnetic field and washing by the buffer. Lastly, the cells combining with microbeads were eluted through the column and collected.

### 2.6. MSC and PBMC Coculture

MSCs cocultured with PBMCs were set up in 12-well plates. Briefly, in the control groups, MSCs were cocultured with PBMCs (2 × 10^5^ cells/well) at the ratio of 1 : 1, 1 : 10, and 1 : 100 for 48 h. Based on the different effects of different SDF-1*α* concentrations on MSCs from the above procedure, SDF-1*α* of 0.1 *μ*g/mL was chosen to stimulate MSCs (2 × 10^5^ cells/well) for 24 h, then the stimulated MSCs were also cocultured with PBMCs at the ratio of 1 : 1, for 48 h in the experimental groups. The culture medium was supplied with 25 ng/mL of CD40L and 200 U/mL of IL-2 (PeproTech, USA). Add 50 ng/mL of PMA, 500 ng/mL of Ionomycin, and 2 *μ*M of Monensin (Sigma, USA) into the coculture system during the last 6 hours. After coculture, the Bregs were collected and stained with anti-CD19, anti-CD24, anti-CD38, and anti-IL-10; then, the Breg generation was examined by using flow cytometry.

### 2.7. Enzyme-Linked Immunosorbent Assay (ELISA)

MSCs were coculture with the B cells isolated from PBMCs. The supernatant level of IL-10 in the medium was measured by ELISA (kits from R&D Systems Inc., USA) after being cultured for 48 h. In another group, after being incubated with 0.1 *μ*g/mL of SDF-1*α* for 24 h, the MSCs were cocultured with B cells for 48 h, and the supernatant level of IL-10 in the medium was also detected. All reagents, standards, samples, and experimental procedures were prepared according to the manufacturer's instructions. Finally, 50 *μ*L of stop solution was added to each well, and absorbance was detected at 450 nm with an automatic ELISA analysis apparatus.

### 2.8. Western Blotting

MSCs were washed with ice cold PBS and harvested in lysis buffer containing proteinase inhibitors. Proteins were separated on sodium dodecyl sulfate polyacrylamide gels (SDS-PAGE, Zeye, Shanghai, China) and transferred onto polyvinylidene difluoride membranes. The membranes were blocked with TBS-T buffer containing 5% nonfat dry milk and incubated overnight at 4°C with primary antibodies against cyclin A, cyclin B, Bax, cleaved caspase-3, and GAPDH (Heidelberg, Germany), followed by washing and incubating with horseradish peroxidase- (HRP-) conjugated secondary antibodies for 2 h at room temperature. The chemiluminescence signal was detected by the use of the ChemiDoc™ MP System (Bio-Rad Laboratories, USA).

### 2.9. Reverse Transcription-Quantitative Polymerase Chain Reaction (RT-qPCR)

MSCs (2.0 × 10^5^) were seeded in 12-well plates, with the medium containing 0.1 *μ*g/mL of SDF-1*α* or AMD3100 (5 *μ*M, Sigma), the CXCR4-specific antagonist. After the MSCs were cultured for 24 h, total RNA was extracted from MSCs using a TRIzol reagent (Invitrogen, USA) according to the manufacturer's instructions. Isolated RNA was reverse-transcribed using a PrimeScript RT Reagent Kit (TAKARA, Japan). The quantity of miR-133 was detected by RT-qPCR using the FastStart Universal SYBR Green Master (Roche, Switzerland); the primers (Shengong, China) used for miR-133 are as follows: sense, 5′-TTGGTCCCC TTCAACC-3′; anti-sense, 5′-GTGCAGGGTCCGAGGT-3′. U6 was used as an internal standard, and the quantifications of the relative gene were calculated by the comparative Ct method: ΔΔCT = ΔCT (test) − ΔCT (calibrator).

### 2.10. Transient Transfection

MSCs (1.0 × 10^4^) were seeded in 12-well plates and transfected with miR-133 inhibitor or negative control using Lipofectamine 2000 (Invitrogen, USA) according to the manufacturer's protocols. The transfected MSCs were treated with 0.1 *μ*g/mL SDF-1*α* for 24 h, and then, cell vitality, cell apoptosis, and cocultured assay were performed. The following primers (Shengong, China) were used for the transfection: miR-133 inhibitor primers, 5′-ACAGCTGGTTCTTGGGACCAAACAGCTGGTTCTTGG-GACCAAACAGCTGGTTCTTGGGACCAAACAGCTGGTTCTTGGGACCAAACAGCTGGTTCTTGGGACCAA-3′; NC primer, 5′-TTCTCCGATGCGTCACGTTTTTCTCCGATGCGTCACGTTTTTCTCCGATGCGTCACGTTTTTCTCCGATGCGTCACGTTTTTCTCCGATGCGTCACGTTTTTCTCCGATGCGTCACGTTT-3′.

### 2.11. The Isolation of Exosomes from MSCs

The MSCs (1.0 × 10^5^) were cultured in 10 cm dishes. When the cell density of MSCs is 70%, replenish with 10 mL fresh complete DMEM medium with exosome-depleted FBS. MSCs were cultured for another 24 hours, then the medium was collected, centrifuged at 300 g for 5 minutes under 4°C to deplete cells, and then centrifuged at 600 g for 5 minutes under 4°C to deplete debris. To further deplete debris, the medium will be centrifuged at 1000 g for 15 minutes under 4°C. Further, the medium was collected and centrifuged at 100,000 g for 2 hours under 4°C; the exosomes will be visible as a pellet at the bottom of the tube. The exosome-depleted medium will also be collected for further experiments. The pellet was washed with PBS and centrifuged at 100,000 g for another 2 hours under 4°C. The pellet was collected and resuspended in 200 *μ*L PBS. The exosomes can be stored under 4°C for one week. To test the effects of MSC-derived exosomes on B cells, 20 *μ*L exosomes will be added into 1 mL medium to culture B cells.

### 2.12. Statistical Analysis

Experimental data were analyzed using SPSS version 21.0 (SPSS Munich, Germany). Values are presented as means ± SD. Statistical analysis was performed using one-way variance analysis to compare data among three or more groups and Student's paired *t* test to compare data between two groups, the Dunnett *t* method was used for multiple comparisons.

## 3. Results

### 3.1. Characterization of Human UC-MSCs

The cells exhibited a spindle-shape and adhered to the culture dishes closely at passage 3. The cells expressed positive for CD105, CD44, and CD49, but negative for CD34 and CD45 [30]. The differentiation potential of UC-MSCs was demonstrated by the differentiation into adipocytes and osteoblasts (Supplementary 1).

### 3.2. MSCs Can Promote Breg Differentiation from Peripheral B Cells of ITP

**Figure 1:**
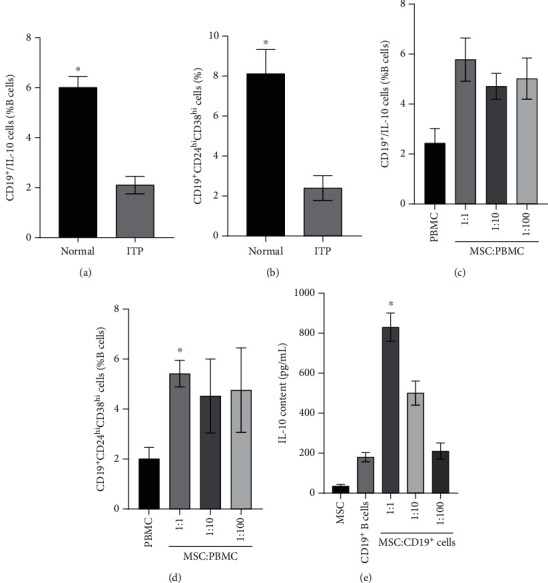
The effects of MSCs in promoting the Breg differentiation. (a) The percentages of CD19^+^CD24^hi^CD38^hi^ B cells in periphery B cells from healthy person and ITP patients. (b) The percentages of IL-10-producing CD19^+^ B cells in periphery B cells from healthy person and ITP patients. (c, d) Coculture MSCs with PBMCs at the ratio of 1 : 1 can significantly increase the percentage of CD19^+^CD24^hi^CD38^hi^ B cells and B10 cells. (e) The concentration of IL-10 in medium promoted notably while coculture CD19^+^ B cells with MSC at the ratio of 1 : 1. ^∗^*P* < 0.05.

Compared to normal controls, lower percentages of both the CD19^+^CD24^hi^CD38^hi^ B cells (8.12 ± 1.21% vs. 2.40 ± 0.62%) and IL-10-producing B cells (6.02 ± 0.44% vs. 2.11 ± 0.35%) were found in B cells from the ITP patient (Figures 1(a) and 1(b), Supplementary 2), revealing the impairment of Bregs in ITP patients. To determine whether UC-MSCs could induce the Breg generation from peripheral B cells of ITP, we found that the MSCs significantly promoted the generation of CD19^+^CD24^hi^CD38^hi^ B cells (2.01 ± 0.46% vs. 5.42 ± 0.53%) and IL-10-producing B cells (2.44 ± 0.58% vs. 5.78 ± 0.86%) (Figures 1(c) and 1(d)).Cocultured MSCs with B cells can increase the IL-10 level, especially at the ratio of 1 : 1 (180.05 pg/mL ± 23.252 pg/mL). Meanwhile, the lower concentration (35.231 ± 8.601 pg/mL) of IL-10 was detected, when the MSCs were cultured individually (Figure 1(e)). These data suggest that human UC-MSCs have the ability to regulate Breg differentiation.

### 3.3. SDF-1*α* Affects the Proliferation, Apoptosis, and Survival of UC-MSCs

**Figure 2:**
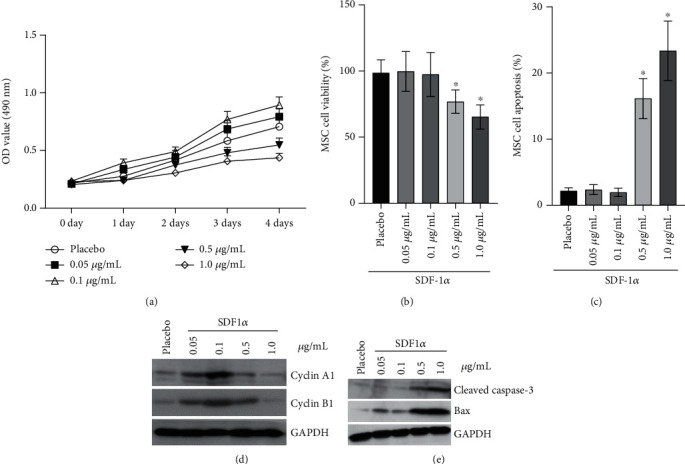
SDF-1*α* affects the survival of MSC by regulating the level of cyclin A1, cyclin B1, caspase-3, and Bax. (a) The effects of different doses of SDF-1*α* on the proliferation of MSCs. (b, c) The effects of different doses of SDF-1*α* on the cell viability and cell apoptosis of MSCs. The viability rate and apoptotic rate had no difference at the concentrations of 0.05 *μ*g/mL and 0.1 *μ*g/mL SDF-1*α*; however, 0.5 *μ*g/mL and 1.0 *μ*g/mL SDF-1*α* could decrease the viability rate and increase the apoptotic rate (2.19 ± 0.48% vs. when compared to the control). (d, e) The effects of different doses of SDF-1*α* on the expression of cyclin A1, cyclin B1, caspase-3, and Bax in MSCs. ^∗^*P* < 0.05.

When UC-MSCs were incubated with different concentrations of SDF-1*α*, the results showed that, compared to the control, the cell proliferating curve of MSCs increased obviously at the concentrations of 0.05 *μ*g/mL and 0.1 *μ*g/mL of SDF-1*α* (Figure 2(a)) but had no difference in MSC viability (98.53 ± 10.06% vs. 99.71 ± 15.13% vs. 97.42 ± 16.70%) (Figure 2(b)) and apoptotic rate (2.19 ± 0.48% vs. 2.37 ± 0.77% vs. 1.96 ± 0.64%) (Figure 2(c)). However, compared to the control, the higher concentrations of SDF-1*α* (0.5 *μ*g/mL and 1.0 *μ*g/mL) could inhibit the proliferation (Figure 2(a)) and the viability (98.53 ± 10.06% vs. 76.81 ± 8.90% vs 65.35 ± 9.12%) (Figure 2(b)) but could increase the apoptotic rate (2.19 ± 0.48% vs. 16.14 ± 3.02% vs. 23.37 ± 4.5) (Figure 2(c)) of UC-MSCs.

Western blotting was performed to investigate the cell cycle regulatory proteins (cyclin A1/B1) and apoptosis-related proteins (cleaved caspase-3/Bax). SDF-1*α* at the lower concentrations could increase the expression of cyclin A1/B1 (Figure 2(d)); however, the expression of cleaved caspase-3/Bax could be promoted at the higher concentrations (Figure 2(e)). So, we select 0.1 *μ*g/mL of SDF-1*α* for the next related research.

### 3.4. SDF-1*α* Is Involved in the Regulation of UC-MSCs on the Differentiation of Bregs in ITP

**Figure 3:**
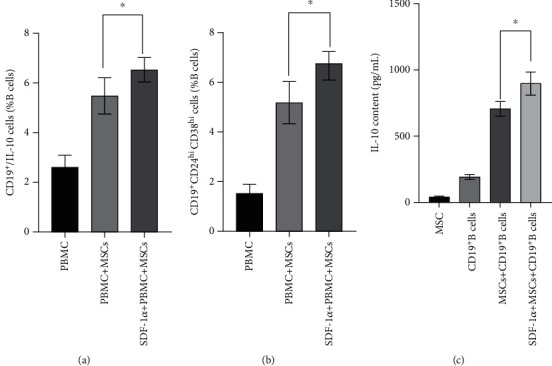
SDF-1*α* pretreatment enhances the property of MSCs in promoting the Breg differentiation. (a, b) 0.1 *μ*g/mL SDF-1*α* promotes the effects of MSCs on IL-10-producing CD19^+^ B cells or CD19^+^CD24^hi^CD38^hi^ cell differentiation. (c) After being treated with 0.1 *μ*g/mL SDF-1*α*, MSCs can increase the secretion of IL-10 from B cells further (897 ± 87 pg/mL vs. 706 ± 55 pg/mL). ^∗^*P* < 0.05.

After being cocultured with the UC-MSCs, the percentage of B10 and CD19^+^/CD24^hi^/CD38^hi^ cells in CD 19^+^ B cells of ITP elevated (2.61 ± 0.48% vs. 5.48 ± 0.73% and 1.52 ± 0.38% vs. 5.18 ± 0.85%) signally (Figures 3(a) and 3(b), Supplementary 3).

Furthermore, compared to the non-SDF-1*α*-stimulated UC-MSC group, B10 and CD19^+^/CD24^hi^/CD38^hi^ cells increased much more (5.48 ± 0.73% vs. 6.53 ± 0.49% and 5.18 ± 0.58% vs. 6.58 ± 0.49%) when the UC-MSCs were incubated with 0.1 *μ*g/mL of SDF-1*α* (Figures 3(a) and 3(b), Supplementary 3).

Compared to the cultures of CD19^+^ B cells (192.67 ± 18.93 pg/mL) and UC-MSCs (42.38 ± 5.725 pg/mL) singly, the concentration of IL-10 in the medium of the cocultured group was promoted (706.33 ± 55.59 pg/mL) notably; when CD19^+^ B cells cultured with the UC-MSCs were pretreated with 0.1 *μ*g/mL of SDF-1*α*, the concentration of IL-10 in the coculture medium would increase (897.74 ± 86.52 pg/mL) further (Figure 3(c)).

### 3.5. SDF-1*α*/CXCR4 Participates in the Expression of miR-133 in UC-MSCs

**Figure 4:**
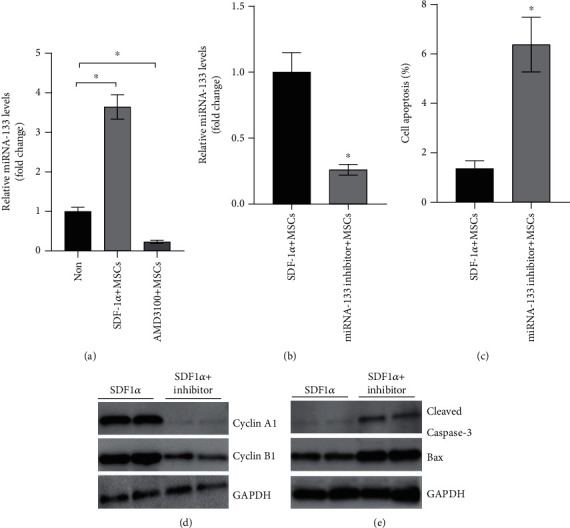
SDF-1*α* regulates the survival of MSCs on B cells through miR-133. (a) The expression of miR-133 in MSC was increased in the presence of 0.1 *μ*g/mL SDF-1*α* but was inhibited after treating with the AMD3100. (b) The effects of miR-133 inhibitor on the expression of miR-133 in MSCs. (c) Transfected with the miR-133 inhibitor, the apoptotic rate of MSCs increased significantly. (d, e) miR-133 inhibitor influences the expression of cyclin A1, cyclin B1, cleaved caspase-3, Bax, and in MSCs. ^∗^*P* < 0.05.

As SDF-1-CXCR4 signaling has an effect on microRNA expression [31], we next assessed the miR-133 level in UC-MSCs after being incubated with the SDF-1*α*. The expression of miR-133 in the UC-MSCs pretreated with 0.1 *μ*g/mL of SDF-1*α* was elevated (1.000 ± 0.108 vs. 3.643 vs. 0.310), while when being treated by AMD3100, the receptor antagonists of CXCR4, the expression of miR-133 in UC-MSCs was remarkably reduced (1.000 ± 0.108 vs. 0.230 vs. 0.041) (Figure 4(a)).

### 3.6. miR-133 Plays a Key Role in SDF-1*α* Controlling UC-MSC Proliferation and Survival

The transfection efficiency of the miR-133 inhibitor was about 70%, which was evaluated by comparing the difference between the bright field and the fluorescence field of the transfected MSCs (Supplementary 4). The expression of miR-133 was remarkably reduced (1.000 ± 0.150 vs. 0.260 vs. 0.041) compared to the US-MSCs transfected by the miR-133 inhibitor, meaning the inhibitor successfully reduced the miR-133 level (Figure 4(b)). Apoptosis of US-MSCs was detected to study the influence of miR-133 on the function of US-MSCs. First, Annexin V/PI staining was performed to detect the apoptosis of the US-MSCs transfected by the miR-133 inhibitor after being cultured for 48 h, and compared to the control, the apoptotic rate of the transfected-MSCs increased (1.36 ± 0.32% vs. 6.38 ± 1.10%) (Figure 4(c)); then, Western blot was performed, and compared to the control, the cell cycle regulatory proteins (cyclin A1/B1) were decreased (Figure 4(d)), but the expression of apoptotic factors cleaved caspase-3/Bax of US-MSCs was increased (Figure 4(e)) after being transfected with the miR-133 inhibitor.

### 3.7. The Induction of Bregs of ITP by UC-MSCs Depends on miR-133

**Figure 5:**
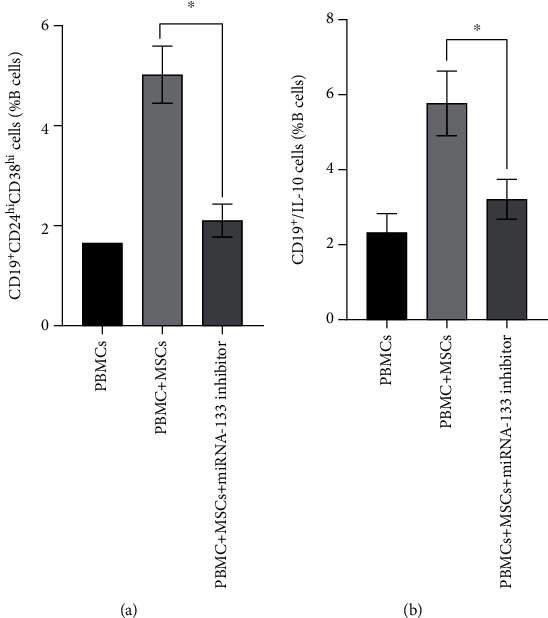
SDF-1*α* regulates the effects of MSCs on B cells through changing the miR-133 level. (a) Transfected by miR-133 inhibitor, the ability of MSCs to facilitate CD19^+^CD24^hi^CD38^hi^ CD19^+^ B cells was depressed. (b) Transfected by miR-133 inhibitor, the ability of MSCs to facilitate IL-10-producing CD19^+^ B cells was depressed. ^∗^*P* < 0.05.

To determine whether miR-133 plays an important role in Breg differentiation induced by UC-MSCs, PBMCs of ITP were cocultured with the US-MSCs transfected by the miR-133 inhibitor for 48 h, and Bregs were detected; the results exhibit that B10 cells differentiated from ITP decreased (3.21 ± 0.54% vs. 5.78 ± 0.86%) evidently compared to the nontransfected group (Figure 5(a), Supplementary 5), and correspondingly, CD19^+^/CD24^hi^/CD38^hi^ cells differentiated from ITP decreased (2.1 ± 0.33% vs. 5.02 ± 0.57%) evidently (Figure 5(b), Supplementary 5).

### 3.8. SDF-1*α* Facilitates MSCs to Promote Breg Differentiation through Exosomal miR-133

**Figure 6:**
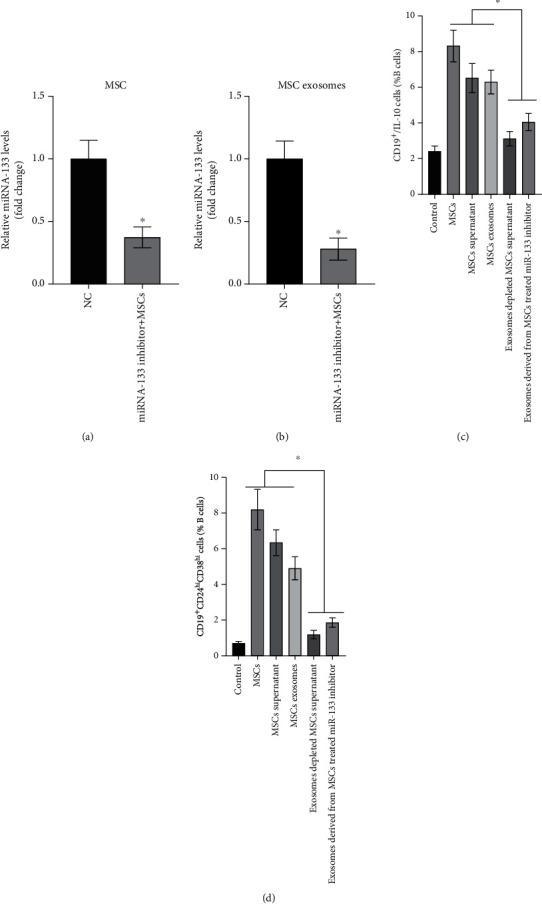
miR-133 mediates the effects of MSCs on B cells dependent on exosome-based delivery. (a, b) Transfected with the miRNA-133 inhibitor, the miRNA-133 level was significantly depressed in MSCs or exosomes. (c, d) When PBMCs are cultured with the MSCs, MSC supernatant, or MSC exosomes, the percentage of CD19^+^CD24^hi^CD38^hi^ CD19^+^ B cells and IL-10-producing CD19^+^ B cells will increase, but this phenomenon was reversed when the MSCs were pretreated with the miRNA-133 inhibitor or the MSC supernatants were depleted of exosomes. ^∗^*P* < 0.05.

miR-133 exits in MSCs and MSC-derived exosomes, and the levels can be suppressed (1.000 ± 0.152 vs. 0.373 ± 0.084% and 1.000 ± 0.143% vs. 0.280 ± 0.088%) by the miR-133 inhibitor (Figures 6(a) and 6(b)). To directly verify whether miR-133 produced by MSCs exerts its roles in an exosome-dependent manner, we used MSCs, complete MSC supernatant, exosome-depleted MSC supernatant, MSC-derived exosomes, and MSC-derived exosomes from MSCs transfected by the miR-133 inhibitor to treat ITP patient-derived PBMCs. Results showed that MSCs, complete MSC supernatant, and MSC-derived exosomes can promote CD19^+^CD24^hi^CD38^hi^ CD19^+^ B cells (0.700 ± 0.101 vs. 8.200 ± 1.135 vs. 6.343 ± 0.722 vs. 4.907 ± 0.645) and IL-10-producing CD19^+^ B cells (2.403 ± 0.306 vs. 8.320 ± 0.887 vs. 6.520 ± 0.814 vs. 6.297 vs. 0.667) in ITP-derived CD19^+^ B cells, while the exosome-depleted MSC supernatant did not show similar effects (B10: 2.403 ± 0.306 vs. 3.117 ± 0.400, CD19^+^CD24^hi^CD38^hi^ CD19^+^ B cells: 0.700 ± 0.101 vs. 1.190 ± 0.24). Moreover, the miR-133 inhibitor can largely reverse the effects of MSC-derived exosomes on CD19^+^CD24^hi^CD38^hi^ CD19^+^ B cells (0.700 ± 0.101% vs. 1.860 ± 0.265%) and IL-10-producing CD19^+^ B cell (2.403 ± 0.306% vs. 4.05 ± 0.49%) generation (Figures 6(c) and 6(d), Supplementary 6–7).

## 4. Discussion

There are no specific intracellular or cell surface markers of Bregs yet [3]; however, IL-10 is a potent negative immunomodulatory factor, so the IL-10-secreting B cells are described as Bregs that have the similar immunosuppressive effects as Tregs [32]. Scholars further described the human Bregs within the CD19^+^CD24^hi^CD38^hi^ immature transitional B cells that this subset has the ability to reduce CD4^+^ T-cell activation via production of the IL-10 and CD80/CD86 pathway [33, 34]. Bregs are potent suppressive lymphocytes for maintaining peripheral tolerance and abating pathogenic immune responses [2]. The CD19^+^CD24^hi^CD38^hi^ subset produced less IL-10 and had reduced suppressive activity, suggesting the deficiency of Bregs in SLE [35]. Li et al. had found that the Bregs are deficient in active disease, and therapy that raises platelet counts also rescues the underachieving B-cell subset [4]. Our results showed the notable reduction of CD19^+^CD24^hi^CD38^hi^ and IL-10-expressing (B10) cell subsets in peripheral blood of ITP, further verifying the abnormal Bregs in ITP [3]. This implies that the decrease of Bregs can lead to the overactivation of autoreactive cells and cause or aggravate the platelet destruction of ITP.

MSCs can not only directly restrain proliferation of the effector B cells [11–13, 36] but also induce differentiation of the CD19^+^CD5^+^ B cell subset of patients with graft versus host disease, which secretes IL-10, thus being recognized as Bregs [18]. After being cocultured with UC-MSCs, we found that the reduced proportion of both CD19^+^CD24^hi^CD38^hi^ and B10 cells of ITP increased significantly, suggesting UC-MSCs can induce immune tolerance in ITP treatment by mediating B cell subgroup changes. However, because of the obstacles in migration and survival of MSCs, there still remain many difficulties for MSC treatment. In great part, the therapeutic effect of MSCs depends on the retention time in the body and tissue [37]. As a chemokine secreted by MSCs, SDF-1*α* can modulate angiogenesis and stem cell recruitment in regenerative process of MSCs [38] and can direct MSC migration in response to inflammatory and/or hypoxic stimuli [39]. It has been demonstrated that SDF-1*α* and its specific receptor CXCR-4 play key roles on MSC migration and survival [40]. Therefore, we observed the effect of different concentrations of SDF-1*α* on UC-MSCs and also found that different concentrations of SDF-1*α* led to different proliferations, survival, and apoptosis results of UC-MSCs and that the lower doses of 0.05 *μ*g/mL and 0.1 *μ*g/mL of SDF-1*α* can promote the proliferation of UC-MSCs, while the higher doses of 0.5 *μ*g/mL and 1.0 *μ*g/mL of SDF-1*α* can promote the apoptosis but inhibit the proliferation and reduce the survival rate of UC-MSCs [29]. However, the mechanism is not clear, so further research is needed to elucidate such differences. Next, 0.1 *μ*g/mL concentration of SDF-1*α* was selected to study whether SDF-1*α*/CXCR4 can directly affect the induction of Bregs by UB-MSCs. The results showed that the SDF-1*α*-stimulated UB-MSCs can further upregulate the proportion of Bregs in ITP, and the IL-10 content in the supernatant also increased much more. These suggest that 0.1 *μ*g/mL of SDF-1*α* could be used as preconditioning for UB-MSC treatment to enhance the immune regulatory effect in ITP.

MicroRNAs (miRNAs) are a series of single-stranded short nucleotides that are involved in gene expression and regulation after transcription in eukaryotic cells and are very important in the realization of many biological functions [41]. The increased expression of miRNAs can enhance the tolerance of MSCs to hypoxia, while knocking down miRNAs will increase the apoptosis rate of MSCs under hypoxia stimulation [42]. Among a large number of miRNAs, miRNA-133 is a kind of miRNA that participates in a variety of cell functions and has an important influence on the proliferation, as well as differentiation, of MSCs [43]. Chen et al. [23] found that the rats transplanted with miR-133-overexpressing MSCs displayed more improved cardiac function after acute myocardial infarction. Therefore, we supposed that SDF-1*α* enhances the cell function by elevating the miR-133 expression in MSCs. So, we detected the miRNA-133 level with 0.1 *μ*g/mL of SDF-1*α* in the control of UC-MSCs. First, we found that SDF-1*α* can mediate the elevation of miRNA-133 in MSCs and lead to less apoptosis. The role of the SDF-1*α*/CXCR4 axis in the survival and function of MSCs has been achieved by influencing miRNA expression [44], and we also found that when treated with AMD3100, the inhibitor of CXCR4, the expression of miRNA-133 in MSCs decreased prominently. Second, the apoptotic rate of MSCs transfected with the miRNA-133 inhibitor significantly increased, and the apoptin-cleaved caspase-3 and Bax in MSCs also increased, while the cyclins in MSCs decreased. These confirm that miRNA-133 plays a key role in controlling the proliferation and survival of UC-MSCs by SDF-1*α*. We further assess the immunological function of MSCs after depressing the miR-133 level, and the results showed that the expression of miR-133 in UC-MSCs was prevented by transfecting the miRNA-133 inhibitor. Compared to the nontransfected UC-MSCs, when miR-133 inhibitor transfected UC-MSCs cocultured with PBMCs of ITP, the induction of B10 cells decreased obviously; correspondingly, the induction of CD19^+^/CD24^hi^/CD38^hi^ B cells also decreased remarkably. These results indicate that the reduction of miRNA-133 in UC-MSCs leads to the decreased differentiation of Bregs. On the one hand, it can be explained that the ability of SDF-1*α* to promote the proliferation and survival of MSCs is achieved by regulating miRNA-133 expression; on the other hand, it also proves that SDF-1*α* enhances the expression of miRNA-133 by interacting with CXCR4, thus promoting the differentiation of Bregs in ITP.

As the Transwell was used to coculture the MSCs with the PBMCs, the cellular interactions should be induced by the soluble mediators. Exosomes are small membrane vesicles participating in intercellular communication and can realize their function by transmitting the exosomal miRNAs [45]. We demonstrated that SDF-1*α* can elevate the miRNA-133 expression of MSCs, as well as the exosomal miRNA-133 secreted by MSCs. The depletion of exosomes in the culture supernatant resulted in the significant reduction of Bregs. This result indicates that exosomal miRNA-133 secreted by MSCs can increase the polarization of Bregs. Although MSCs have been shown to inhibit immune cell activation, reduce inflammatory cytokines, and induce autoimmune tolerance and have already been used as treatment methods [46], the exact regulating mechanism on how SDF-1*α* controls MSCs in inducing Breg differentiation of ITP still requires more and intensive research, especially involving with miRNA-133 expression.

## 5. Conclusions

UC-MSCs could effectively enhance the decreased proportion of Bregs from ITP; different doses of SDF-1*α* have different effects on the proliferation, apoptosis, and survival of UC-MSCs, and at appropriate concentrations, SDF-1*α* may further promote the proliferating and survival ability of UC-MSCs and improve the production of Bregs induced by UC-MSCs through controlling the miRNA-133 expression in the exosomes, which displays a novel solution in improving the immunoregulatory capacity of MSCs for ITP management.

## Data Availability

The data used to support the findings of this study are available from the corresponding author upon request.

## Supplementary Materials

Supplementary Materials Supplementary 1: the morphology of the MSC and the FACS analysis for the surface markers. Supplementary 2: the original picture of FACS in Figures 1(a) and 1(b). Supplementary 3: the original picture of FACS in Figures 3(a) and 3(b). Supplementary 4: morphology of MSCs observed under inverted fluorescence microscope. Supplementary 5: the original picture of FACS in Figures 5(a) and 5(b). Supplementary 6-7: the original picture of FACS in Figures 6(c) and 6(d).
