# Supplementary material for: SDF-1α Facilitates Mesenchymal Stem Cells to Induce Regulatory B Cell Differentiation from Patients with Immune Thrombocytopenia
Source: Stem Cells Int. 2021 Nov 8;2021:3254488. doi: 10.1155/2021/3254488 (PMC8592740; doi:10.1155/2021/3254488)
Supplement: Supplementary Materials — Supplementary 1: the morphology of the MSC and the FACS analysis for the surface markers. Supplementary 2: the original picture of FACS in Figures 1(a) and 1(b). Supplementary 3: the original picture of FACS in Figures 3(a) and 3(b). Supplementary 4: morphology of MSCs observed under inverted fluorescence microscope. Supplementary 5: the original picture of FACS in Figures 5(a) and 5(b). Supplementary 6-7: the original picture of FACS in Figures 6(c) and 6(d). [file 3254488.f1.zip › Supplemental Figure 1.pdf]

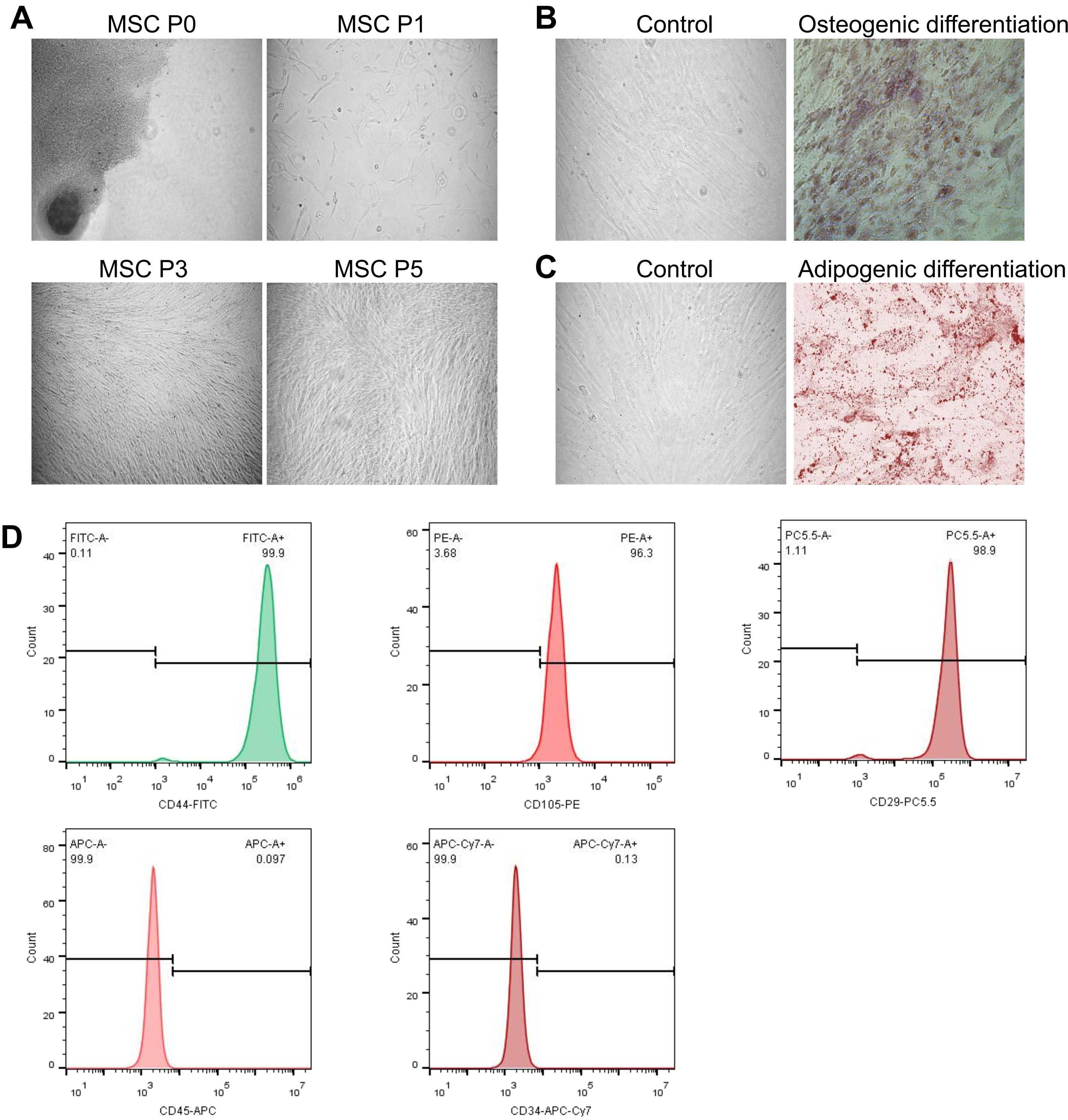

Supplymentary Figure 1 The Morphology of the MSC and the FACS analysis for the surface markers (A).The characterization of UC-MSC from passage 0 to passage 5 (B).After in the osteogenic differentiation medium, UC-MSC at passage 3 was stained with alkaline phosphatase (C). After in the adipogenic differentiation medium, UC-MSC at passage 3 was stained with Oil Red O.(D）The surface markers were detected by flow cytometry, and the cells expressed positive for CD105, CD44 and CD49, but negative for CD34 and CD45.
