# Supplementary material for: SDF-1α Facilitates Mesenchymal Stem Cells to Induce Regulatory B Cell Differentiation from Patients with Immune Thrombocytopenia
Source: Stem Cells Int. 2021 Nov 8;2021:3254488. doi: 10.1155/2021/3254488 (PMC8592740; doi:10.1155/2021/3254488)
Supplement: Supplementary Materials — Supplementary 1: the morphology of the MSC and the FACS analysis for the surface markers. Supplementary 2: the original picture of FACS in Figures 1(a) and 1(b). Supplementary 3: the original picture of FACS in Figures 3(a) and 3(b). Supplementary 4: morphology of MSCs observed under inverted fluorescence microscope. Supplementary 5: the original picture of FACS in Figures 5(a) and 5(b). Supplementary 6-7: the original picture of FACS in Figures 6(c) and 6(d). [file 3254488.f1.zip › Supplemental Figure 2.pdf]

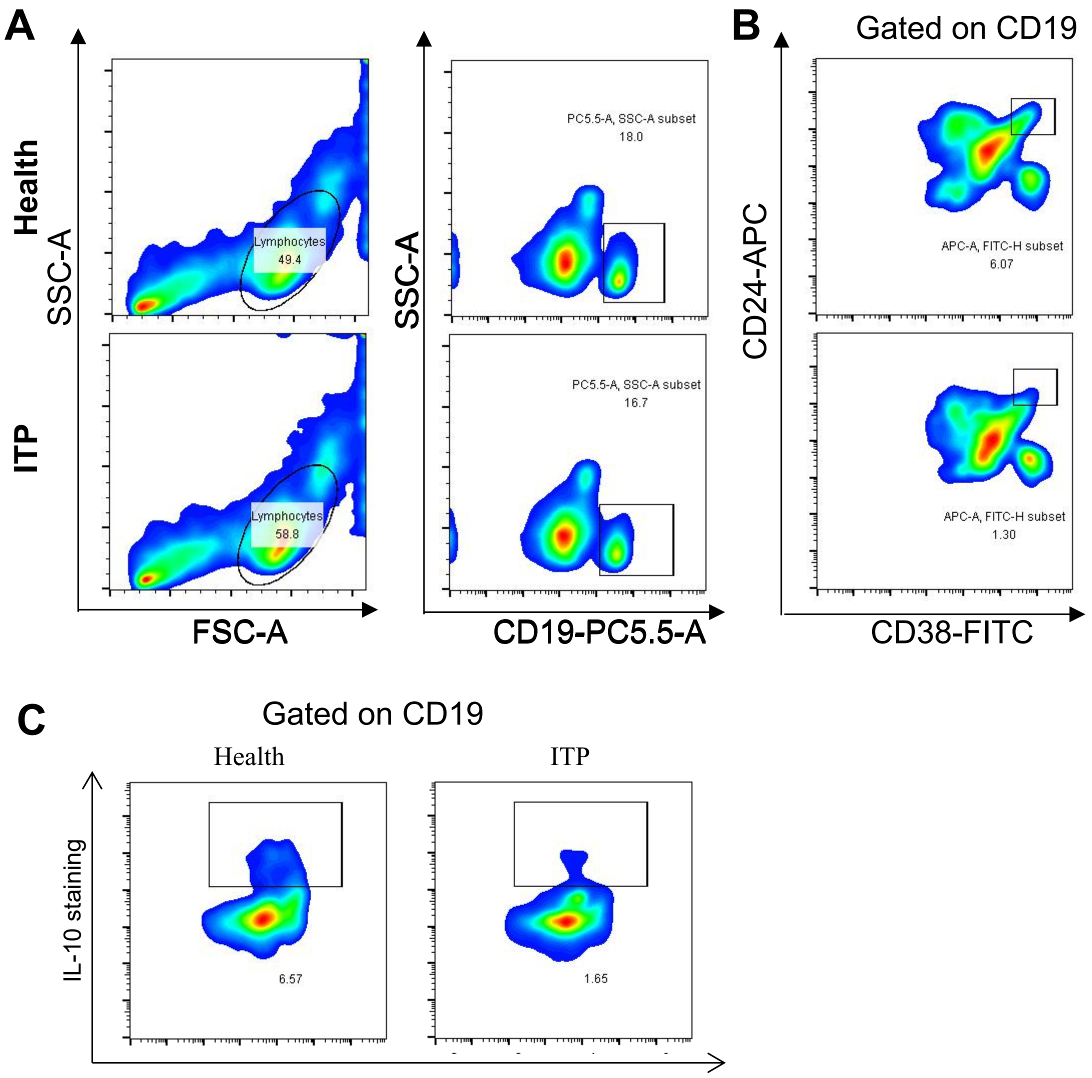

Supplymentary Figure 2.FACS analysis for Bregs form the ITP/health sample.
(A) B cells binding with PC5.5 labelled anti-CD19 were selected.Next,the cells(B) binding with APC labelled anti-CD24 and FITC labelled anti CD 38 were selected form CD-19 B cells.In additon, IL-10 staining CD 19 B cells(C) were selected.The quantity of these cells were tested by flow cytometry.
