# Supplementary material for: SDF-1α Facilitates Mesenchymal Stem Cells to Induce Regulatory B Cell Differentiation from Patients with Immune Thrombocytopenia
Source: Stem Cells Int. 2021 Nov 8;2021:3254488. doi: 10.1155/2021/3254488 (PMC8592740; doi:10.1155/2021/3254488)
Supplement: Supplementary Materials — Supplementary 1: the morphology of the MSC and the FACS analysis for the surface markers. Supplementary 2: the original picture of FACS in Figures 1(a) and 1(b). Supplementary 3: the original picture of FACS in Figures 3(a) and 3(b). Supplementary 4: morphology of MSCs observed under inverted fluorescence microscope. Supplementary 5: the original picture of FACS in Figures 5(a) and 5(b). Supplementary 6-7: the original picture of FACS in Figures 6(c) and 6(d). [file 3254488.f1.zip › Supplemental Figure 3.pdf]

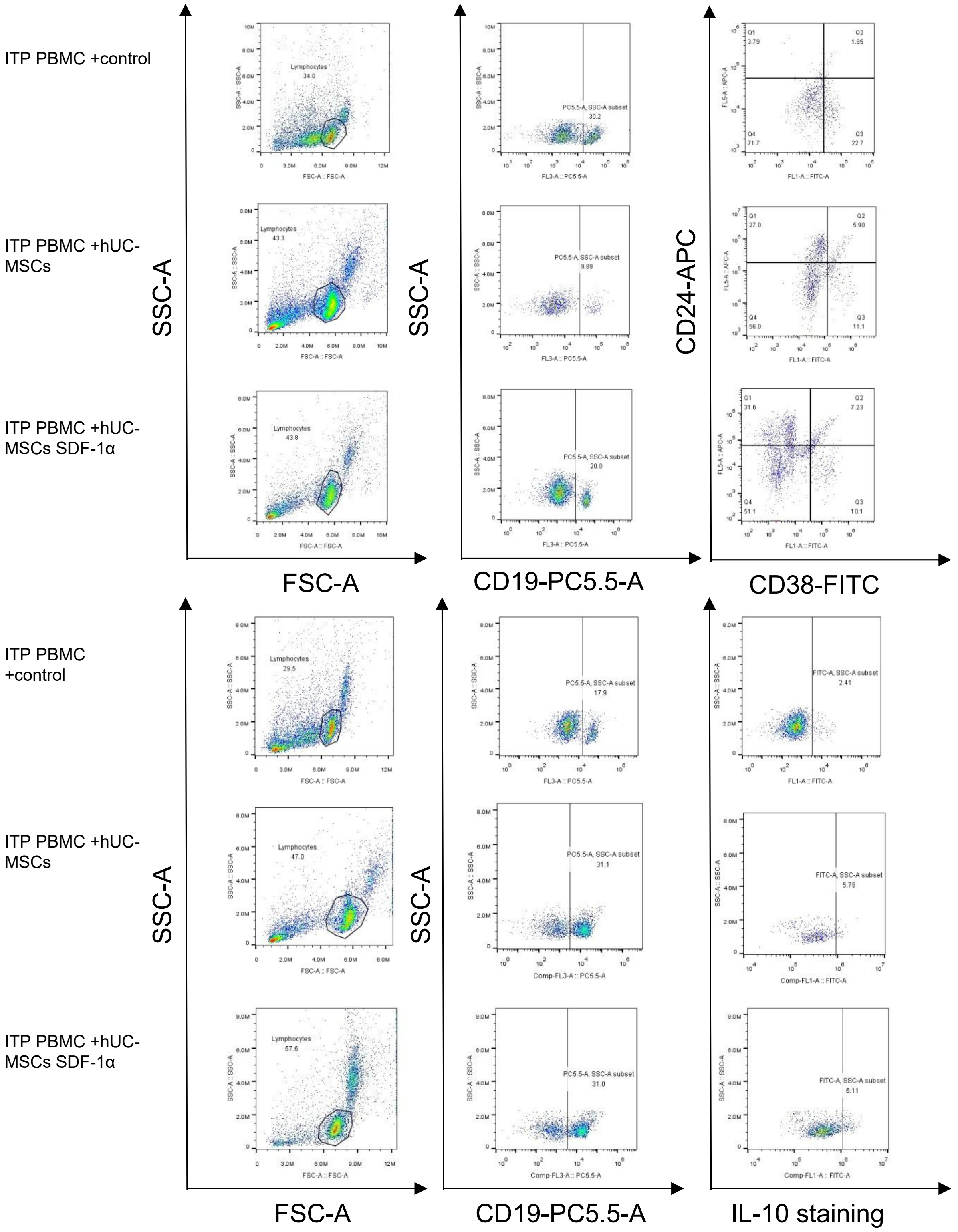

Supplymentary Figure 3. SDF-1α enhanced the ability of MSC to induce Bregs.
PBMC was co-culture with the MSC at the ratio of 1:1 for 48h.The culture medium was supplied with 25 ng/Ml of CD40L, and 200 U/Ml of IL-2.50 ng/mL of PMA, 500 ng/mL of Ionomycin and 2 μM of Monensin was added into the co-culture system during the last 6 hours. The FACS analysis showed that the proportion of Bregs increased. After PBMC was co-culture with the MSC simulated for 24h in the medium containing 0.1μg/mL SDF-1α , the amount of Bregs from PBMC increased futher.
