# Supplementary material for: SDF-1α Facilitates Mesenchymal Stem Cells to Induce Regulatory B Cell Differentiation from Patients with Immune Thrombocytopenia
Source: Stem Cells Int. 2021 Nov 8;2021:3254488. doi: 10.1155/2021/3254488 (PMC8592740; doi:10.1155/2021/3254488)
Supplement: Supplementary Materials — Supplementary 1: the morphology of the MSC and the FACS analysis for the surface markers. Supplementary 2: the original picture of FACS in Figures 1(a) and 1(b). Supplementary 3: the original picture of FACS in Figures 3(a) and 3(b). Supplementary 4: morphology of MSCs observed under inverted fluorescence microscope. Supplementary 5: the original picture of FACS in Figures 5(a) and 5(b). Supplementary 6-7: the original picture of FACS in Figures 6(c) and 6(d). [file 3254488.f1.zip › Supplemental Figure 7.pdf]

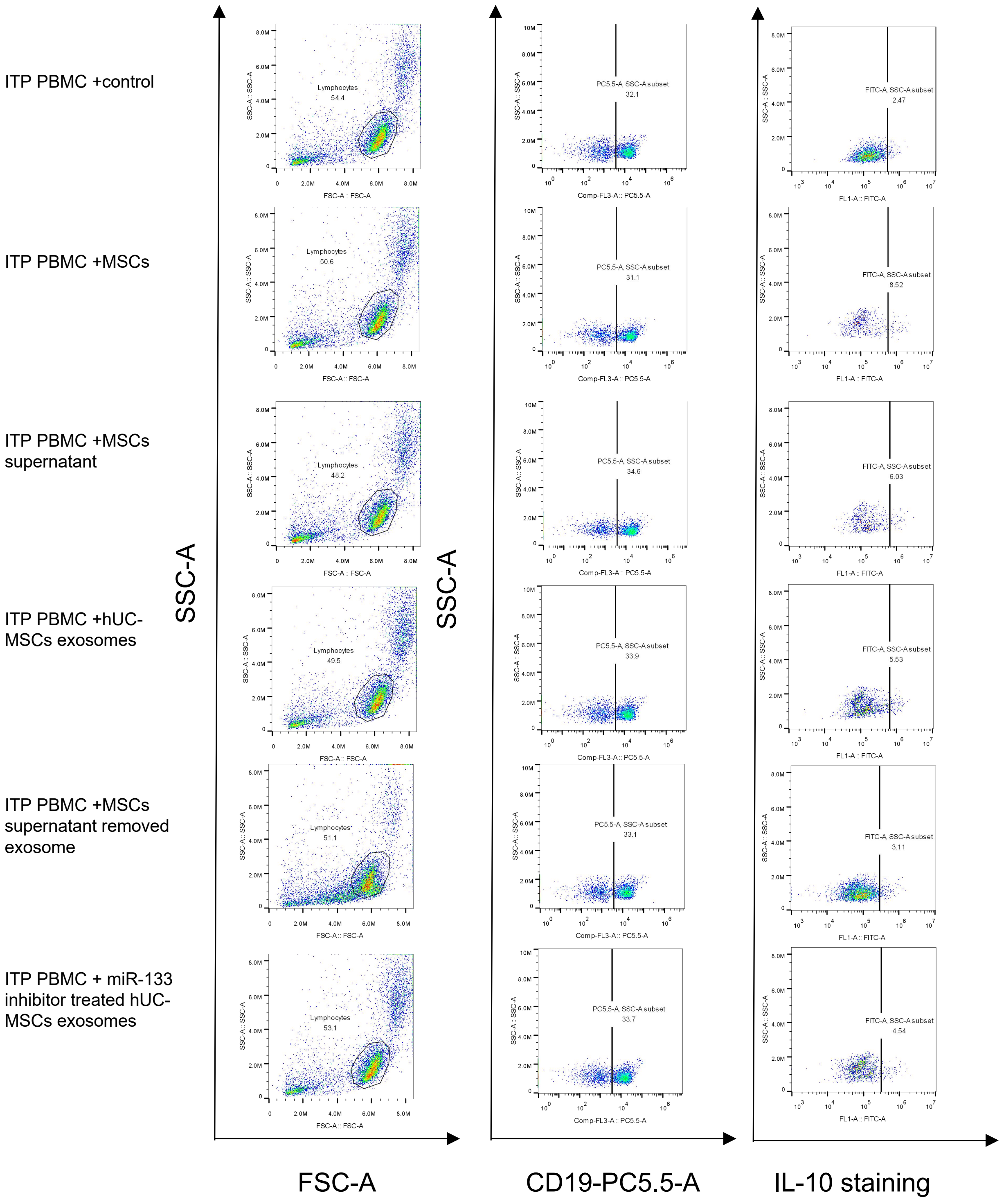

Supplymentary Figure 7.MSC induced CD19⁺/IL-10 Bregs through exosomes.
The CD19⁺/IL-10 Bregs was analyzed by FACS after co-culture with the MSC,MSC supernatant, MSC exosomes, exosomes depeleted MSC supernatant or the exosomes derived from miRNA-inhibitor tansfected MSCs
